# Point Mutations in the Transmembrane Region of the Clic1 Ion Channel Selectively Modify Its Biophysical Properties

**DOI:** 10.1371/journal.pone.0074523

**Published:** 2013-09-18

**Authors:** Stefania Averaimo, Rosella Abeti, Nicoletta Savalli, Louise J. Brown, Paul M. G. Curmi, Samuel N. Breit, Michele Mazzanti

**Affiliations:** 1 Dipartimento di Bioscienze, Università degli Studi di Milano, Milan, Italy; 2 Department of Molecular Neuroscience, University College London, Institute of Neurology, London, United Kingdom; 3 Department of Chemistry and Biomolecular Sciences, Macquarie University, Sydney, Australia; 4 School of Physics, University of New South Wales, Sydney, Australia; 5 St Vincent’s Centre for Applied Medical Research, St Vincent’s Hospital, Sydney, Australia; University G. D'Annunzio, Italy

## Abstract

Chloride intracellular Channel 1 (CLIC1) is a metamorphic protein that changes from a soluble cytoplasmic protein into a transmembrane protein. Once inserted into membranes, CLIC1 multimerises and is able to form chloride selective ion channels. Whilst CLIC1 behaves as an ion channel both in cells and in artificial lipid bilayers, its structure in the soluble form has led to some uncertainty as to whether it really is an ion channel protein.

CLIC1 has a single putative transmembrane region that contains only two charged residues: arginine 29 (Arg29) and lysine 37 (Lys37). As charged residues are likely to have a key role in ion channel function, we hypothesized that mutating them to neutral alanine to generate K37A and R29A CLIC1 would alter the electrophysiological characteristics of CLIC1. By using three different electrophysiological approaches: i) single channel Tip-Dip in artificial bilayers using soluble recombinant CLIC1, ii) cell-attached and iii) whole-cell patch clamp recordings in transiently transfected HEK cells, we determined that the K37A mutation altered the single-channel conductance while the R29A mutation affected the single-channel open probability in response to variation in membrane potential.

Our results show that mutation of the two charged amino acids (K37 and R29) in the putative transmembrane region of CLIC1 alters the biophysical properties of the ion channel in both artificial bilayers and cells. Hence these charged residues are directly involved in regulating its ion channel activity. This strongly suggests that, despite its unusual structure, CLIC1 itself is able to form a chloride ion channel.

## Introduction

Chloride intracellular channel 1 (CLIC1) is a member of a family of intracellular ion channels [1,2,3,4,5]. It is a 241-amino acid protein, homologous to the carboxy-terminal domain of p64 (CLIC5B), which was the first characterized member of the CLIC family. CLIC5B was initially purified from bovine kidney cortex membrane on the basis of chloride channel activity after reconstitution of the purified protein into artificial lipid membranes [6,7].

CLIC1 is a metamorphic protein that can shift between two or more different stable conformations [8,9,10]. It exists largely as a soluble intracellular protein but under appropriate conditions can insert into lipid membranes [4,11,12,13]. Under reducing conditions, CLIC1 is monomeric and structurally homologous to the GST superfamily, with a redox active site resembling glutaredoxin [14,15]. Upon oxidation, CLIC1 undergoes a major structural rearrangement of the thioredoxin-like N-domain, resulting in a non-covalent dimeric form [9]. This may be an intermediate form in the transition between soluble and membrane-inserted CLIC1 [16].

The structure of soluble CLIC1 does not resemble that of any other ion channel protein. However, bioinformatics and biophysical studies suggested that CLIC1 has a single putative transmembrane domain (PTM) sufficient to cross the lipid bilayer [2,9]. Biophysical studies have determined that the PTM enters the lipid bilayer [16], and models based on fluorescence energy transfer data suggest that between six and eight CLIC1 proteins assemble into an oligomer to create an ionic chloride conduction pathway [17]. Indeed, despite its unusual structure, several groups have previously published data indicating that CLIC1 and other CLICs can function as ion channels. We and others have shown that purified soluble CLIC1 inserts into an artificial bilayer, mediating single-channel chloride currents with the same characteristics as those recorded in patch clamp experiments from CLIC1 transfected cells [18,19,20]. Nevertheless, some concern still exists as to whether CLIC1 can or cannot act as an ion channel [21].

The transmembrane domain is important in defining the biophysical properties of any ion channel. Indeed, very often mutations in these regions affect specific features of an ion channel, such as conductance, gating or ion selectivity [22,23]. In order to assess whether CLIC1 represents a chloride selective ion channel *per se*, we have performed point mutational studies on the CLIC1 PTM sequence. Such a mutational approach has been widely used to test the biophysical properties of other ion channels [22,24,25,26].

The PTM region of CLIC1 encompasses residues 24 to 46 and contains only two charged residues [27]. Charged residues within a membrane spanning segment are likely to be involved in regulating ion flow. For this reason, we have produced two mutant forms of CLIC1 by independently replacing these two charged residues, Arg29 and Lys37, with alanine (R29A and K37A, respectively). These two CLIC1 mutants were then evaluated for ion channel activity using three different approaches: artificial lipid membranes (Tip-Dip method), cell-attached and whole-cell electrophysiological recordings of transiently transfected HEK cells. The results of these studies show that substituting the only charged residues in the PTM of CLIC1 with neutral alanine modifies the electrophysiological properties of the CLIC1 ion channel. The changes seen *in vitro* are commensurate with the observed electrophysiological properties observed in cells. The fact that mutating PTM residues alters channel properties provides the strongest evidence to date, that despite its unusual structure, CLIC1 has all the hallmarks expected of an ion channel.

## Materials and Methods

### Cell culture and transfection

Human embryonic kidney (HEK) 293 cells were maintained in Advanced D-MEM (Gibco-Invitrogen, Carlsbad, CA) supplemented with 10% FBS (fetal bovine serum), 2 mM glutamine, 100 units/ml penicillin and 100 µg/ml streptomycin. The cells were cultured at 37°C in a 5% CO_2_ humidified incubator. HEK 293 cells were transfected with the DNA of N-terminal FLAG-tagged human CLIC1 (WT) or its K37A or R29A mutants cloned in pIRES2-EGFP vector (Clontech Laboratories Inc., San Jose, CA). The pIRES2-EGFP plasmid used for these experiments contains the internal ribosome entry site (IRES), which permits both the gene of interest and the EGFP coding region to be translated as separate proteins from a single mRNA. HEK 293 cells were plated in petri dishes at 70% confluence. After 12h the cells were transiently transfected with Superfect Reagent (Qiagen GmbH, Hilden, Germany) according to the manufacturer’s instructions. Cells were used 48 h after the beginning of transfection.

### Expression and purification of *E. coli* expressed CLIC1

CLIC1 and its mutants were expressed as GST-CLIC1 fusion proteins in *E. coli* BL21(DE3) using the pGEX-4T-1 vector. The recombinant CLIC1 was separated from the GST purification tag by enzymatic cleavage and purified as previously described [15,20].

### Electrophysiology

Single-channel recordings from lipid bilayers were obtained using the Tip-Dip method [15,20]. In brief, patch clamp pipettes (Garner Glass 7052) were made using a P97 Sutter Instruments puller (Novato, CA), coated with Sylgard (Dow Corning, Midland, MI) and fire-polished to a tip diameter of 1–1.5 µm and 5–7 megaohm resistance. The same solution (140 mM KCl, 10 mM Hepes, pH 6) was used both in the bath and in the pipette. As soon as the pipette tip reached the bath solution, a phospholipid monolayer (phosphatidylcholine, Avanti Polar Lipids, Inc., Birmingham, AL) was spread on the surface. The electrode was repeatedly passed through the surface of the solution until the pipette resistance rose above 5 gigaohm. Purified recombinant wild type, R29A or K37A CLIC1 proteins were then added to the bath at a final concentration of 2 µg/ml.

Patch clamp electrophysiology was performed in cell-attached or whole cell configuration in HEK cells transiently expressing CLIC1 using standard methods as previously reported [28]. GFP fluorescence, detected by a Zeiss Axiovert 100 fluorescent microscope, allowed recognition of transfected cells for electrophysiological experiments. The bath solution was (in mM): 90 NaCl, 40 TEACl, 2 CaCl_2_, 2 MgCl_2_, 10 HEPES, 10 Glucose, pH 7.35. The cell-attached electrode was filled with (in mM): 127.5 N-methyl-D-Glucamine-Cl, 5 KCl, 10 TEACl, 2.5 CaCl_2_, 1 MgCl_2_, 5 4-Aminopyridine, 10 HEPES, 10 Glucose, pH 7.35. For perforated patch experiments, the electrode contained (in mM) 20 TEACl, 120 TEACH _3_SO_4_, 10 HEPES, 10 Glucose, pH 7.2. A similar solution was used for current clamp experiments to measure changes in the membrane resting potential. Amphotericin B (Sigma, St Louis, MO, USA) was used for voltage and current-clamp experiments at a concentration of 60 µg/ml. This antibiotic forms pores in the plasma membrane enabling the flow of monovalent ions.

For whole cell experiments, the intracellular solution was (in mM): 20 KCl, 120 K-aspartate, 10 HEPES, 1 MgCl_2_. The potassium channel blocker tetraethyl-ammonium (TEA 40 mM; Sigma, St Louis, MO, USA) was used to minimize the contribution of these ions. Under these conditions, the average resting membrane potential was -22 ± 1.8 mV (n = 20) compared to -40 ± 2.4 mV (n = 10) recorded using a more physiological bath solution (145 NaCl, 5 KCl, 2 CaCl_2_, 2 MgCl_2_, 10 HEPES 10 Glucose, pH 7.35). To obtain voltage-current relationships (i/V), the cell voltage was held at -50 mV and the current was measured at the end of 800 ms voltage steps from -70 to + 50 mV. A subtraction method using 50 µM indanyloxyacetic acid 94 (IAA94, Sigma, St Louis, MO, USA) a Cl- channel inhibitor, was used to isolate the inhibitor-sensitive current. In our experimental conditions, the theoretical chloride reversal potential was -38 mV, assuming that in a perforated patch configuration using Amphotericin B the intracellular chloride concentration is similar to the one in the pipette solution. We calculated a tip potential of -9.8 mV (PClamp 9, Axon Instruments, Novato, CA) that was added to all the plots regarding whole-cell experiments.

The Axopatch 200 B amplifier and the pClamp 9 acquisition software and Clampfit 9 (both from Molecular Device, Novato, CA) were used to record and analyze both whole-cell and single-channel. Current recordings were digitized at 5 kHz and filtered at 1000 Hz. The single-channel conductance is determined by linear regression of the i/V relationship. Due to the extensive channel flickering, amplitude histograms with a 0.01 pA interval resolution have been useful to determine average channel close and open state. Distribution of events between the two levels has been defined by a threshold of 50% of the channel amplitude obtained from the amplitude histograms. Based on an idealized channel reconstruction the software organizes an event list from which parameters like open time probability or mean open and close time are calculated. Open probabilities and mean open and close time have been calculated over at least 5 minutes of continuous recordings.

Macroscopic conductance *versus* voltage curves (*G*(*V*) curves) were calculated dividing the current-voltage relationship by the driving force (*Vm* -*E*_*Cl*_), where *V*m is the membrane potential and *E*_*Cl*_ the equilibrium potential for Cl^-^, estimated using the Nernst equation. G(V) curves were fitted to one Boltzmann distribution of the form: *G*(*V*) = *G*_*max*_/(1+exp(*z* (*V*_*half*_ -*V*_*m*_ (*F*/*RT*))) where *G*_*max*_ is the maximal *G*; *z* is the effective valence of the distribution; *V*_*half*_ is the half activating potential; F, R and *T* are the usual thermodynamic values.

### Statistical analysis

Electrophysiology data are presented as the mean ± standard errors of the mean. Values obtained from different experiments were tested for statistical differences using one-tail two population t-test for independent samples (OriginLab). Data were considered to be statistically different when p ≤ 0.05. 

## Results

Mutations that alter the transport properties of a channel protein provide insight into channel structure and function. Thus, we assessed the effect of point mutations of the only two positively charged residues in the CLIC1 PTM, Lys37 and Arg29, using three different electrophysiological approaches.

### Electrophysiological properties of WT, K37A and R29A CLIC1 in Tip-Dip configuration

**Figure 1:**
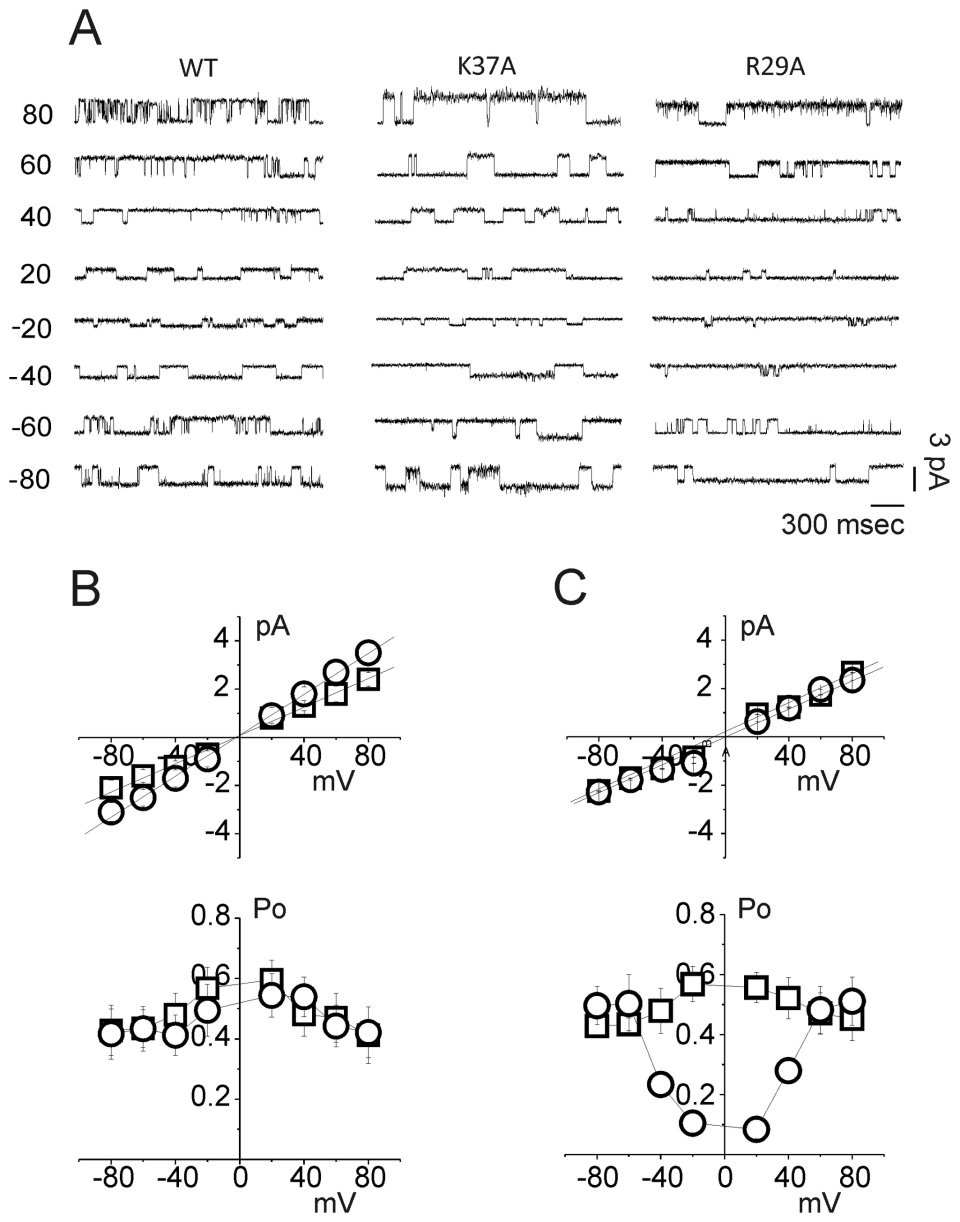
CLIC1 ion channel activity from Tip-Dip bilayer experiments. Current recordings from -80 to +80 mV, 20 mV interval, are shown in (A) for wild type CLIC1 (left), K37A (center) and R29A (right) CLIC1 protein. The upper panel of Figure 1B depicts the single-channel current-voltage data for WT (□) and K37A (○) CLIC1 proteins. The average single-channel conductance differs between WT and K37A, calculated as 30.1 ± 0.2 and 42.4 ± 0.2 pS, respectively (n = 5, p < 0.001). In contrast, the channel open probability of the K37A mutation is very similar to the WT (Figure 1B, lower panel). The current/voltage relationships for both WT (□) and R29A CLIC1 (○) are shown in (C). The single-channel conductance (upper panel), is 30.1 ± 0.2 and 29 ± 0.2 pS for WT and R29A, respectively. The average open probability for WT (□) and R29A mutated protein (○) is shown in the lower panel.

We firstly assessed the single channel properties of purified recombinant wild type (WT) and point mutated recombinant CLIC1 in artificial membranes, using the Tip-Dip method (Figure 1). The WT protein showed ion channel activity with a 33% success rate (20 channel recordings out of 60 trials). Similar to the WT, K37A showed channel activity in 27% of the trials (15 channel recordings out of 55 trials), while the R29A mutant produced channels with a 20% of success rate (11 channel recordings out of 54 trials).

K37A CLIC1 displays a significantly steeper single-channel current-voltage relationship than WT or R29A proteins. The single channel analysis showed that the resulting conductance of K37A was significantly higher than the WT (WT= 30.1 ± 0.2 pS, n = 11 and K37A = 42.4 ± 0.2 pS, n = 5; p < 0.001; Figure 1B, upper panel). Thus, the K37A mutation increased the single channel conductance by 40% compared to the WT. In contrast, the conductance value of R29A CLIC1 (R29A = 29 ± 0.2 pS, n = 6; Figure 1C, upper panel) was indistinguishable from WT.

Using the same methods, we also determined the open probability (Po) of the ion channel as a function of membrane potential for WT, K37A and R29A CLIC1. K37A CLIC1 has a very similar Po to the WT CLIC1 (Figure 1B, lower panel). However, the Po for R29A CLIC1 is much lower than the WT protein at membrane potentials between -40 and +40 mV (Figure 1C, lower panel), while the values do not notably differ from WT CLIC1 for potentials below -50 mV or above +50 mV. These results show that R29A mutation affects the Po in a membrane potential range around 0 mV (i.e. close to the reversal potential for chloride under the conditions of the experiment) [29].

### Untransfected HEK cells do not display CLIC1 associated electrophysiological activity

**Figure 2:**
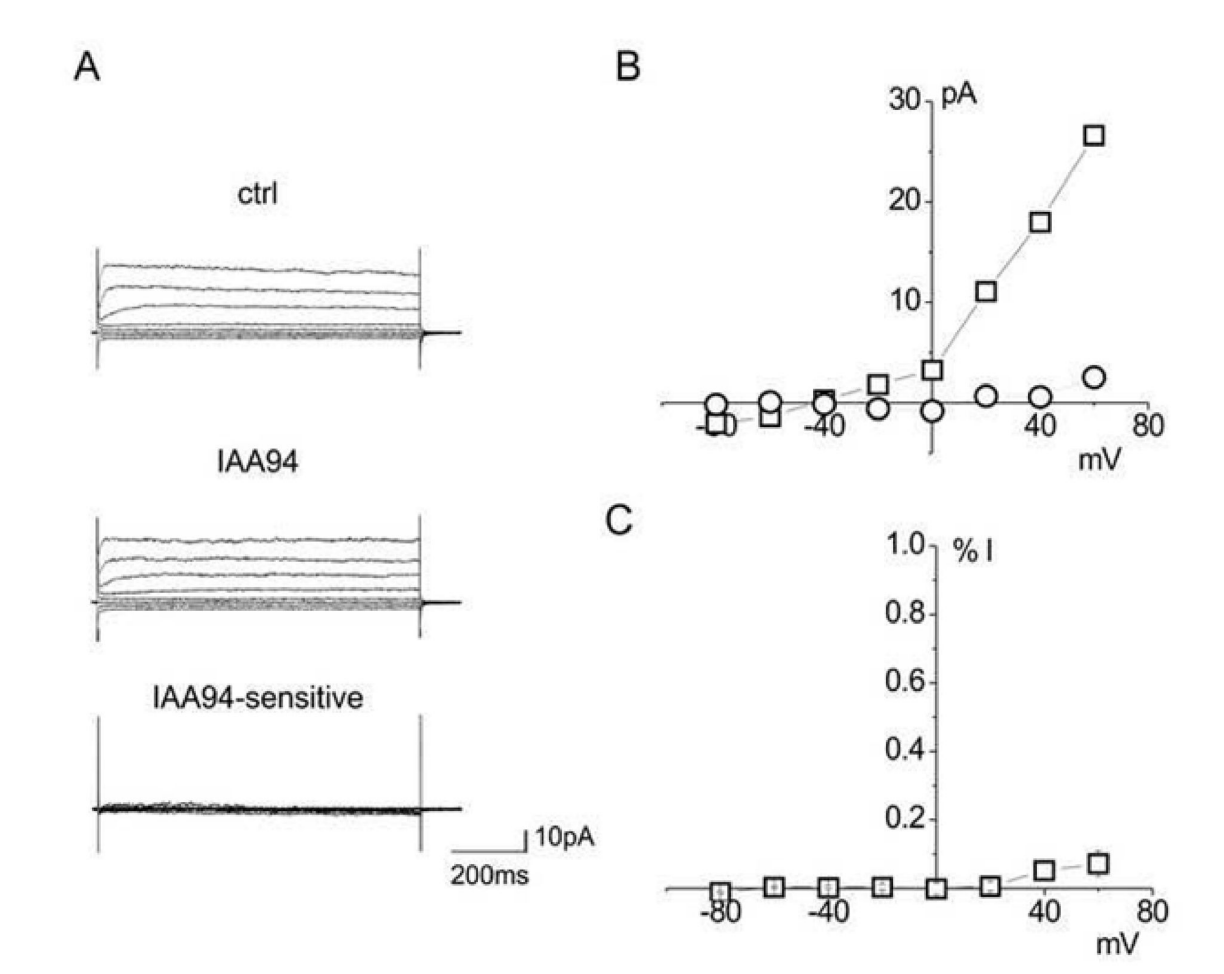
Endogenous CLIC1 is not expressed on the plasma membrane of untransfected HEK cells. (A) Family of currents for an untransfected HEK293 cell at varying applied voltages from -60 to +60 mV with 20 mV increment. Top panel: whole cell current in resting conditions; middle panel: after IAA94 perfusion; lower panel: IAA94-sensitive (CLIC1-mediated) current obtained from subtraction. (B) i/V curve of an IAA94-sensitive current in WT CLIC1 transfected HEK293 cell (□), and in an untransfected HEK293 cell (○). (C) Plot of the average of the IAA94-sensitive current as a percentage of the control current of untransfected HEK cells (n=5). Data are shown as mean ± SEM. CLIC1-mediated current is completely absent in untransfected HEK cells.

In order to enable the investigation of the electrophysiological properties of K37A and R29A CLIC1 in a cell system, we first tested for any interference from endogenous CLIC1 that might be present in our HEK cell line. As a control, we examined the same HEK cells transfected with WT CLIC1. Whole cell current recordings from an untransfected HEK cell being perfused with physiological solution are shown in Figure 2A, upper panel. The whole cell currents remained unaltered upon perfusion with 100 µM IAA94, a known inhibitor of CLIC1 ion channel activity [20,30] (Figure 2A, middle panel). By subtracting the residual current after IAA94 perfusion (Figure 2A, middle panel) from the current recorded in control conditions (Figure 2A, upper panel), we obtained the IAA94-sensitive current, which was negligible (Figure 2A, lower panel). By contrast, transfection with WT CLIC1 results in an IAA94-sensitive current (Figure 2B, squares), whilst at the same time no such current was observed in the untransfected HEK cells (Figure 2B, circles). Further, in these untransfected HEK cells, the average current/voltage plot of the IAA94-sensitive current from five different experiments shows that it is indeed negligible (Figure 2C), confirming that, under our experimental conditions, there is no detectible CLIC1 current in control HEK cells. The absence of a detectable IAA94-sensitive endogenous current makes HEK cells a useful tool for electrophysiological studies concerning the effects of heterologous expression of WT and mutant CLIC1 proteins.

### K37A mutation affects single-channel electrophysiological properties of CLIC1 channel in HEK cells

#### Single channel conductance

**Figure 3:**
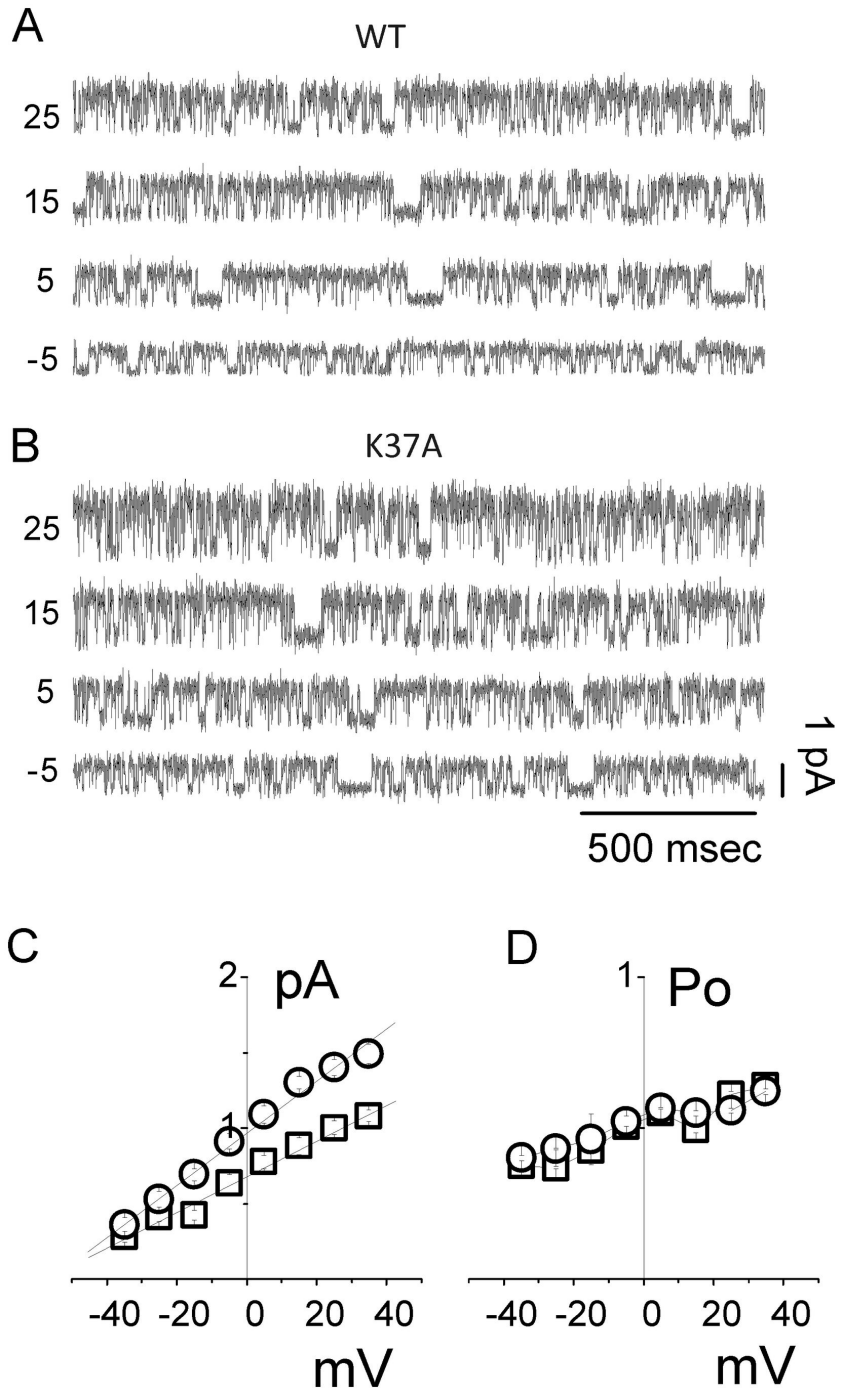
Cell-attached recordings of HEK cells transiently transfected with WT and K37A CLIC1 protein. Single-channel current traces are shown for WT (A) and K37A (B) CLIC1 transfected cells. The membrane voltage was clamped at different values (indicated on the left of each trace) in 10 mV step increments. Single-channel current/voltage plots are shown in (C) for WT (□) and K37A (○). Average single-channel conductance of 12.1 ± 0.6 pS for WT and 17.4 ± 0.8 pS for K37A with an extrapolated reversal potential of -58 ± 0.7 mV and -56 ± 1.2 mV, respectively, was calculated. The open probability obtained at each membrane potential is shown in (D) for WT (□) and K37A (○).

To further investigate the effect of CLIC1 point mutations on its ion channel activity, HEK cells were transiently transfected either with constructs expressing WT or K37A CLIC1. Figure 3 shows an example of a single channel recording of the WT (Figure 3A) and K37A CLIC1 protein (Figure 3B) at four different membrane potentials (from -5 mV to +25 mV, 10 mV steps). We found a significant increase in the conductance for the K37A CLIC1 (Figure 3C; circles) compared to the WT CLIC1 (Figure 3C; squares). The linear fit through the experimental points gives an average conductance of 12.1 ± 0.6 pS (n=5) for WT CLIC1 and 17.4 ± 0.8 pS (n=5) for K37A CLIC1. The increase in conductance for K37A compared to WT CLIC1 is statistically significant (p < 0.01). This increased single channel conductance of K37A in these cell attached experiments is consistent with the increased conductance observed in the Tip-Dip experiments (Figures 3C and 1B, respectively).

The extrapolation of the linear fits of the i/V data to zero current shows average reversal potentials of -58 ± 0.7 mV and -56 ± 1.2 mV for both WT and K37A CLIC1, respectively (Figure 3C; n= 5). This demonstrates that the K37A mutation did not affect ion channel selectivity. Further, the K37A CLIC1 channel open probability did not show any significant difference compared to WT (Figure 3D; WT = 0.38 ± 0.07; K37A = 0.35 ± 0.07; n= 5). This is consistent with the data obtained from the tip-dip experiments where the open probabilities for WT and K37A CLIC1 channels were indistinguishable (Figure 1B).

#### Open and close state kinetics

**Figure 4:**
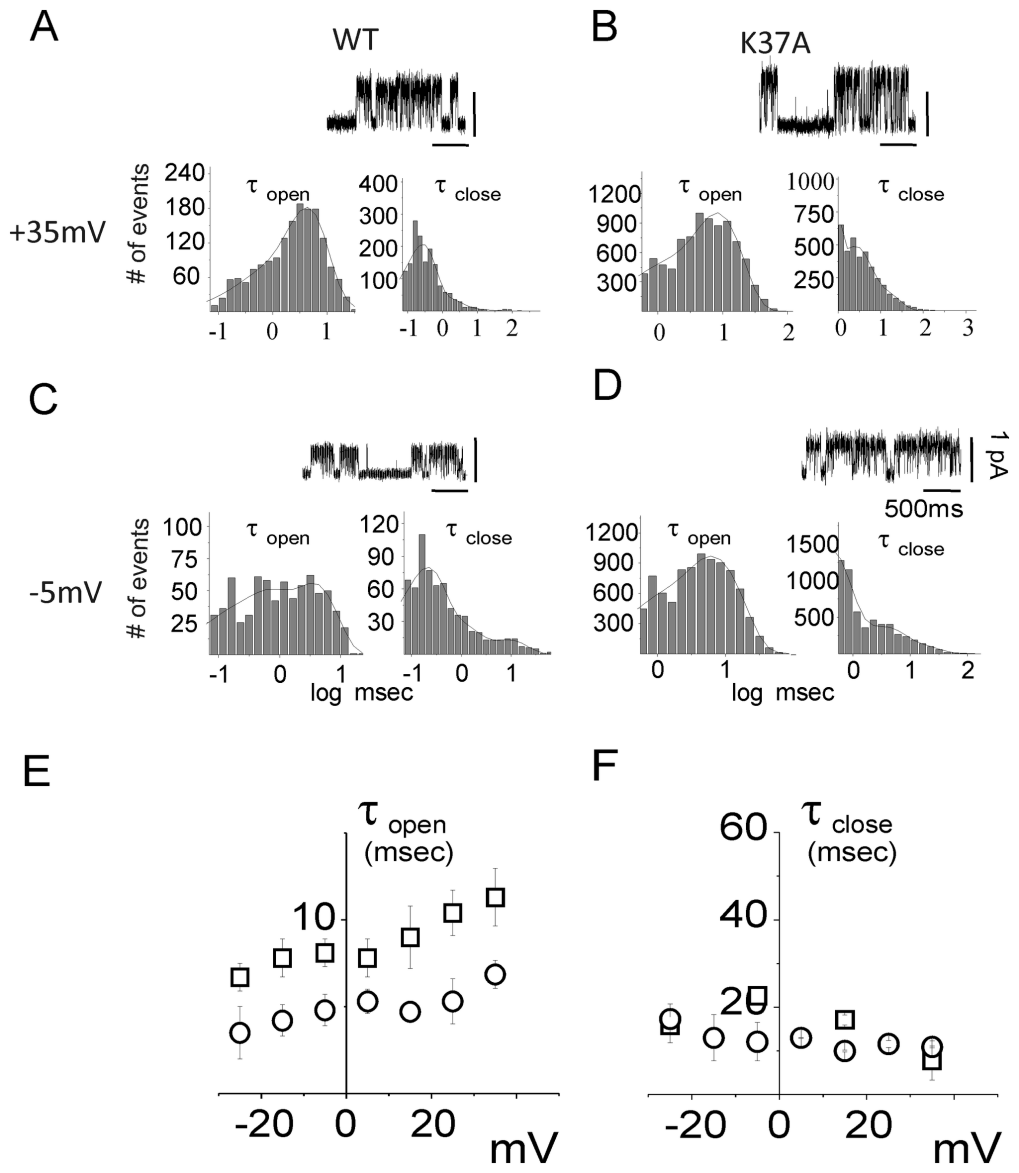
Open (τ_open_) and close (τ_close_) time constants of the WT and K37A CLIC1 ion channel. In panels (A) and (B), membrane potentials were held at +35 mV while panels (C) and (D) concern cell membrane potentials held at -5 mV for WT (A, C) and K37A (B, D) CLIC1 transfected HEK cells. (A) to (D) shows the open (left) and close (right) time distributions for each condition. Four seconds of single channel recordings appear as inserts in the corresponding panels for each condition. The open and close time distribution histograms were fitted by a double exponential decay function and plotted on a semi-logarithmic scale. Panels (E) and (F) depict open and close time distributions as a function of membrane potential for WT (□) and K37A (○) transfected HEK cells.

**Figure 5:**
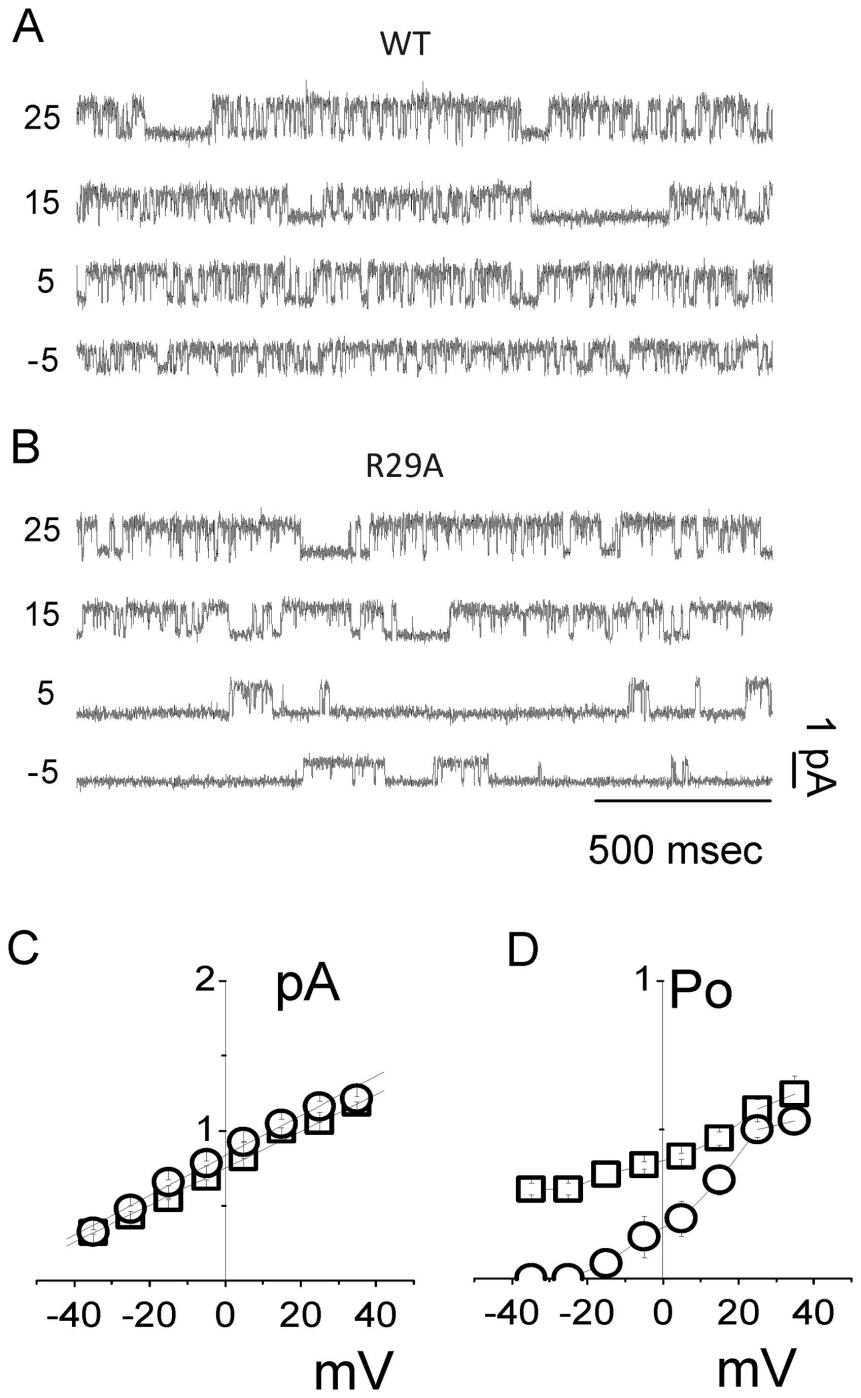
Cell-attached recordings of HEK cells transiently transfected with WT CLIC1 and R29A CLIC1 protein. Single-channel current traces are shown for WT (A) and R29A (B) transfected cells. The voltage steps are from -5 to + 25 mV (10 mV step increment). Single-channel current/voltage plots are shown in (C) for WT (□) and R29A (○). Average single-channel conductance of 12.3 ± 0.1 pS for WT and 13.1 ± 0.3 pS for R29A and an extrapolated reversal potential of -63 ± 0.4 mV and -61 ± 0.6 mV, respectively. The open probability obtained at each membrane potential is shown in (D) for WT (□) and R29A (○).

For a more detailed comparison between the biophysical properties of WT and K37A CLIC1, further analyses of the single-channel kinetics were performed. We have analyzed the open (τ_open_) and close time (τ_close_) distributions at different membrane potentials, from -25 mV to +35 mV, for both WT and K37A CLIC1 (Figure 4). The analysis includes a total of 7 minutes of recording for both WT and K37A in cell-attached single-channel configuration from five different experiments. The open- and close-time distribution histograms were fitted to a double exponential decay function and plotted on a semi-logarithmic scale. We considered only the slower time constant difference since the faster time constant is directly linked to the sample rate and to the cut-off frequency. Panels A to D display examples of open- and close-time analysis at two different membrane potentials: +35 mV (Figure 4A and B) and -5 mV (Figure 4 C and D) for both WT (panels A and C) and K37A (panels B and D). From these data, the calculated average open-time at +35 mV was 11.3 ± 1.7 ms and 6.9 ± 0.8 ms (Figure 4A and B, left panels) and 8.1 ± 0.8 ms and 4.8 ± 0.9 ms at -5mV (Figure 4C and D, left panels) for WT and K37A mutant, respectively. A complete analysis at all the membrane potentials tested shows that the mean open time (τ_open_) for K37A CLIC1 was decreased at every membrane potential (4E; p < 0.01, n= 5), whereas the mean close time (τ_close_) distributions for the WT and the K37A mutant appeared similar (Figure 4F). However, Figure 4 E and F highlights a difference in the mean open time without a corresponding difference in mean close time. This apparent contradiction is explained by the increased channel flickering in K37A CLIC1. Since our comparison only considered the slower exponential fitting (see Methods), τ_open_ decreases for the mutated protein while in contrast, τ_close_ is unaltered for long closing times. Changes in the short timescale events have not been considered in this analysis because they were too dependent on the sample rate and the filter that was used in the experiments.

### Effects of R29A mutation on the single-channel electrophysiological properties of CLIC1 in HEK cells

#### Single channel conductance

In order to characterize the R29A point mutation in a cell system, we expressed the R29A and WT control CLIC1 constructs separately into HEK cells. Figure 5 shows examples of single channel recordings at four different membrane potentials (from -5 mV to +25 mV, 10 mV steps) for HEK cells expressing WT and R29A (5A and 5B, respectively). The average single channel conductance of WT and R29A CLIC1 was statistically indistinguishable (Figure 5C, WT = 12.3 ± 0.1 pS, n=7; R29A = 13.1 ± 0.3 pS, n=5). This is consistent with the Tip-Dip data where the R29A mutation did not affect the channel conductance (Figure 1C).

The data shows similar average reversal potential for both WT and R29A (-63 ± 0.4 mV WT and -61 ± 0.6 mV R29A; n= 5), providing evidence that the mutation is not likely to affect the permeability properties of CLIC1.

#### Open probabilities

There was a marked reduction in the open probability of R29A CLIC1 in the cell-attached recordings of transfected HEK cells, similar to that detected in Tip-Dip recordings. Whilst in WT transfected cells the Po increases at depolarizing membrane potentials (Figure 5D, squares), in R29A transfected cells, the observed Po is smaller than WT below +20 mV and is near zero for membrane potentials < -25 mV (Figure 5D, circles). Above +20mV, R29A Po values resemble those observed in WT CLIC1 expressing cells (Figure 5D; n=4).

Whilst in Tip-Dip experiments (Figure 1), the difference in the open probability is evident for membrane voltages between -50 and +50 mV, in the cell-attached configuration (Figure 5D) the differences in the open probability start at membrane potentials below +30 mV. The open probability (Po) reaches zero at about -15 mV, which is approximately 45 mV above the channel reversal potential. So, as in Tip-Dip experiments, the R29A mutation affects the single channel kinetics at membrane potentials near the reversal potential.

#### Open and close state kinetics

**Figure 6:**
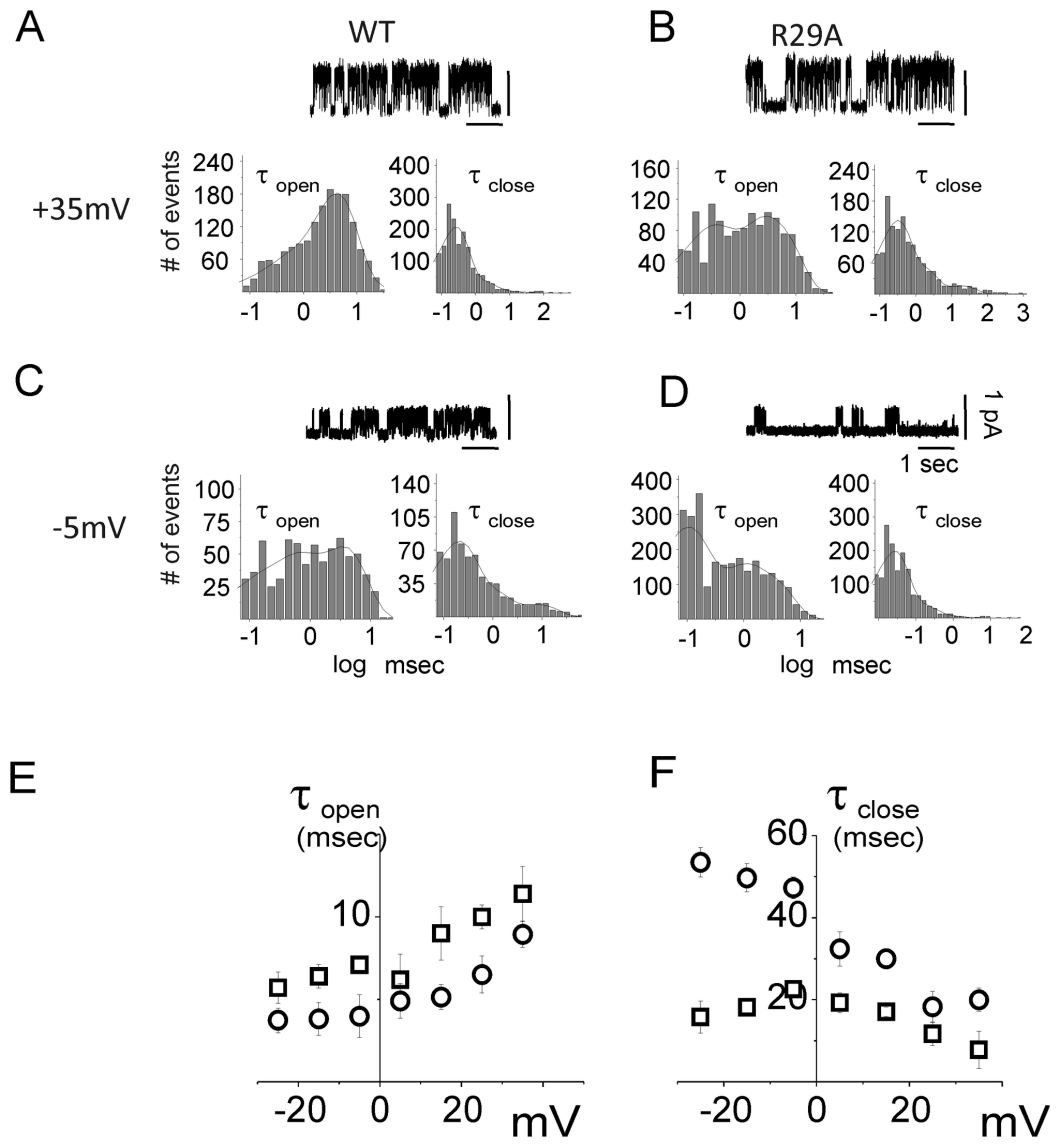
Open (τ_open_) and close (τ_close_) time constants of the WT and R29A CLIC1 ion channel. In panels (A) and (B), membrane potentials were held at +35 mV while panels (C) and (D) concern cell membrane potentials held at -5 mV. The Figure shows open (left) and close (right) time distribution for WT (A, C) and R29A (B, D) CLIC1 transfected HEK cells. Four seconds of single channel recordings appear as inserts in the corresponding panels for each condition. The open and close time distribution histograms were fitted by a double exponential decay function and plotted on a semi-logarithmic scale. Panels (E) and (F) depict open and close time distributions as a function of membrane potential for WT (□) and R29A (○) transfected HEK cells.

In order to further characterize the effect of the R29A mutation on CLIC1 channel properties, we measured the open and close time distributions of R29A CLIC1 at different applied voltages, from -25mV to +35 mV (Figure 6). Examples of this analysis are shown at two specific membrane potentials: +35 mV and -5mV (Figure 6A-D). At +35 mV (Figure 6A and B, left) the open times were 11.4 ± 2.0 and 8.9 ± 0.8 ms for WT and R29A, respectively. At -5mV the open time values were 7.1 ± 0.4 for WT and 4.0 ± 1.3 ms for R29A. These values were not statistically different (n = 4), showing that at both membrane potentials the open times are unaffected by the R29A mutation. However, at -5 mV the R29A CLIC1 displays a marked increase in the close time duration (Figure 6D) compared to the WT (Figure 6C). The average close times were 22.6 ± 1.6 and 48 ± 3 ms for WT and R29A CLIC1, respectively (n = 4, p < 0.01). In contrast, at +35 mV the close time values for WT and R29A CLIC1 are similar, being 8.2 ± 3.5 and 10.6 ± 0.4 ms, respectively. The analysis at all the membrane potentials tested shows that the mean close time is drastically affected by the R29A mutation, in particular at more negative membrane potentials (Figure 6F). Therefore, once the channel reaches its open conformation, the time spent in the open state is only slightly affected by the R29A CLIC1 mutation (Figure 6E). However, in R29A CLIC1 the switch between the closed and open conformation is reduced. Thus, at negative potentials, the overall time spent in the closed conformation is greatly increased.

### Effects of R29A mutation on macroscopic current in CLIC1 transfected HEK cells

Our data indicates that the K37A mutation in CLIC1 causes an increase in the single-channel conductance (Figures 1 and 3). All the other parameters (open probability and channel time constants) remain similar to those seen in WT CLIC1. In whole-cell experiments, the comparison of K37A with WT CLIC1 current would be unreliable because the current amplitude depends on the transfection efficiency, the expression of functional protein and its insertion into the plasma membrane, all of which are unpredictable. However, a change in the current voltage dependence, as shown in the Po analysis for R29A CLIC1 (Figures 1 and 5), would be evident in a whole-cell G/V curve. For this reason, we have investigated whole cell recordings of HEK cells transfected with R29A or WT CLIC1.

**Figure 7:**
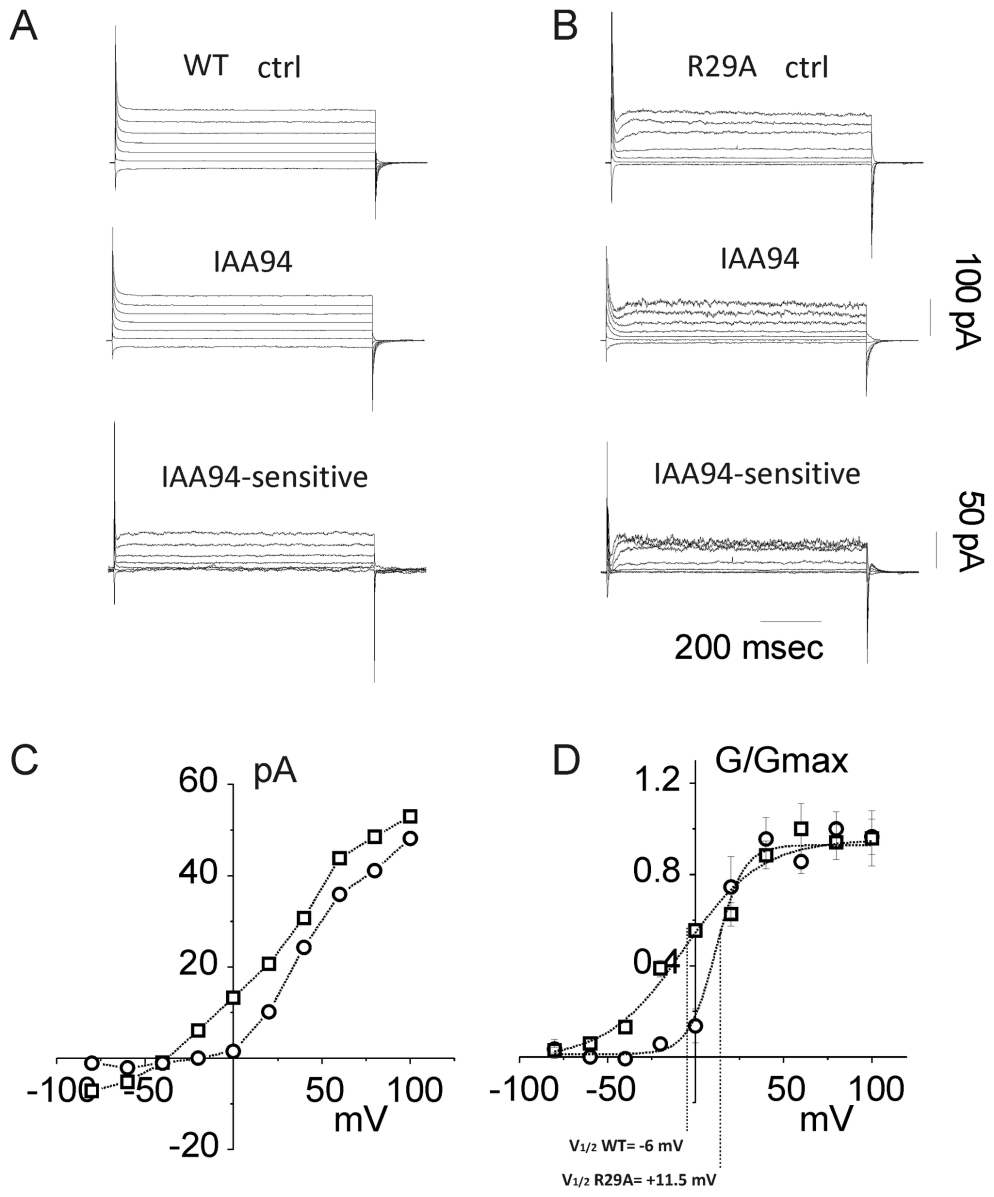
Whole-cell current in R29A transfected HEK cells. Family of whole-currents for WT (A) and R29A (B) transfected HEK cells (top panels). Voltage steps lasting 800 ms from holding potential of -30 mV to membrane potential of -80 to +100 mV (20 mV step increment). The middle panels depict the whole-cell currents after perfusion of 50 µM of IAA94. The bottom panels represent the IAA94-sensitive currents obtained by subtraction of the middle panel current from the upper panel current. Note that the IAA94-sensitive currents are plotted on a different scale with the scale bars on the right hand side of the figure. (C) an example of current/voltage relationship of the IAA94-sensitive current from a WT (□) and a R29A (○) transfected HEK cell. (D) Averaged G/V plots from IAA94-sensitive current of WT (□) and R29A mutant (○), from 5 independent experiments.

Figure 7 shows an example of whole-cell current recordings at different voltage steps from WT (Figure 7A) and R29A (Figure 7B) CLIC1 transfected HEK cells. The currents recorded in control conditions from either WT (Figure 7A, upper panel) or R29A transfected HEK cells (Figure 7B, upper panel) were decreased by 5 minutes perfusion of 100 µM IAA94 (Figure 7A and B, middle panels). We then subtracted the steady state current after IAA94 inhibition from the control current to obtain the IAA94-sensitive CLIC1-mediated current (bottom panels of Figure 7A for WT and 7B for R29A).

Figure 7C depicts the i/V plot of IAA94-sensitive currents showing a decreased current at negative membrane potential for R29A mutant compared to the WT CLIC1 transfected cells. In WT, the IAA-94 sensitive current has an average reversal potential of -42.0 ± 3.6 mV (Figure 7C; n = 5). In R29A transfected cells, we can only determine the zero current since there is no recordable inward current as the current flow ceases between -10 and -15 mV. In order to explain the effect of the R29A mutation on the whole cell current, we analyzed macroscopic conductance (G) to compare Po changes in macroscopic currents from WT and R29A CLIC1 expressing cells. From -30 mV holding potential, we applied 800 ms voltage steps every 20 mV from -80 to +100 mV. The calculated G appeared to be completely saturated by +100 mV. Thus, the normalized averaged (n=5) G for WT and R29A CLIC1 transfected cells (G/Gmax) plotted against membrane potential were fitted by a single Boltzmann equation (Figure 7D). The half activation potential (V_half_) for WT and R29A was -6.56 ± 4.62 mV and 11.56 ± 4.00 mV (n = 5 p < 0.01), respectively, indicating a shift in the voltage dependence towards more positive potentials in R29A transfected cells. The normalized G values were statistically different at applied voltages of -40mV, -20 and 0 mV (n = 5, p < 0.01). However, the values were not statistically different at all the other membrane potentials analyzed. Thus, in keeping with the results obtained in R29A CLIC1, there is a marked decrease of the Po at membrane voltages close to the chloride reversal potential, supporting the view that a single mutation of Arg29 affects the voltage sensitivity of CLIC1, as shown by the Po curves obtained in cell attached experiments (Figure 5D).

## Discussion

Association between mutagenesis of specific residues and alteration of electrophysiological properties of an ion channel has been used to identify key ion channel regions such as the pore, the gate and the selectivity filter of many ion channel proteins [31,32]. In this paper, we have mutated the only two charged residues in the CLIC1 PTM to alanine and observed changes in the conductance (K37A) and changes in the channel open probability (R29A). These observed changes in channel function are consistently seen both in Tip-Dip studies (using artificial bilayers and purified soluble recombinant CLIC1) and in CLIC1 transfected cells studied using both single channel recordings and whole cell currents. Our observations show that each of these residues controls a distinct property of the CLIC1 channel: Lys37 the conductance and Arg29 the open probability. Thus, our results provide strong evidence that CLIC1 acts as an ion channel and confirm that both Lys37 and Arg29 are in a functionally important region of the CLIC1 ion channel.

Although the changes to the electrophysiological properties of the channel produced by the two mutations are consistent in Tip-Dip and cell experiments, there are some differences between the i/V plots from the Tip-Dip experiments (Figure 1B-C) and cell attached recordings (Figures 3C and 5C). The cell-attached experiments show a shift in the reversal potential and a reduced single channel conductance when compared to the Tip-Dip experiments. Regarding the shift of the reversal potential, this is due to the different Cl- concentration used in Tip-Dip (symmetrical 120 mM chloride, E_Cl_ = 0 mV) compared to the cell-attached experiments (internal chloride concentration around 20 mM, external 140 mM, E_Cl_ = -60 mV).

Concerning the differences in the single channel conductance, these have been previously reported for CLIC1 [29] and found to be due to the different chloride concentration used in Tip Dip (equimolar) compared to the chloride concentration in HEK cells. Thus, equimolar chloride concentration results both into a rise in the single channel conductance and a shift in the reversal potential of CLIC1 currents. Despite that, equimolar chloride concentrations have been widely used in artificial bilayer experiments by several groups [33,34,35]. This is because it enables a more accurate analysis and thus helps to better distinguish channel openings at low applied membrane voltages. The dependence of channel properties on ion concentration is not unique to CLIC1. For example, the IK1 potassium channel is regulated by changes in K concentration and the removal of divalent cations from the cytoplasmic side leads not only to an expected shift of the reversal potential, like it happens in all ion channels, but also to an increase in the conductance and elimination of the channel voltage dependence [36,37]. However, whilst the chloride dependence of CLIC1 is the likely explanation for differences between Tip-Dip and cell associated ionic currents, we cannot rule out the possibility of the involvement of other interacting/modifying proteins, second messengers, or differences with lipid composition that are present in cell recordings but missing in Tip-Dip artificial bilayers, where there is only purified recombinant CLIC1 and a simplified artificial lipid cocktail.

Although the structure of the transmembrane form of CLIC1 has not been determined at high resolution, biophysical studies in artificial bilayers support the formation of a multimeric pore containing the CLIC1 PTM as the membrane spanning segment [17]. This is consistent with the recently proposed model of the CLIC1 channel structure by Singh in which Arg29 and Lys37 faced the inner part of the putative pore of CLIC1 [27]. According to this model these two charged residues line the channel pore, extending their charged side chains into the pore region and forming two rings at the top and at the center of the channel. Although this model of the channel pore requires experimental verification, it is consistent with our findings which indicate that Arg29 and Lys37 have important roles in modulating the biophysical properties of the CLIC1 channel.

The increase in CLIC1 single channel conductance resulting from the K37A mutation is an unexpected result. Since a positive charge in chloride channels would be expected to attract anions, the removal of a positive charge might be expected to decrease the conductance. Although we do not know the actual position of Lys37 within the CLIC1 channel pore, using the model proposed by Singh we speculate that this K37A mutation might affect the strength of the interaction of the permeating anions with residues lining the channel pore [33,34]. That is, the removal of the positive charge may reduce the residence time of anions in the channel pore resulting in a faster flow of ions and hence, an increased flow of ions per unit time.

Tip Dip, cell attached and whole cell experiments all demonstrate that the R29A CLIC1 mutation alters the channel open probability, Po. Nonetheless, the Po/V curve for R29A CLIC1 drawn from Tip-Dip experiments has a somewhat different shape from the Po/V curve from transfected cells (compare Figure 1C, bottom panel with Figure 5D). However, if we only consider the data at positive membrane potentials from Tip-Dip experiments, the Po/V plot for the R29A mutant has a sigmoidal relationship similar to the one recorded in cellular systems, with an expected shift towards negative potentials in cell systems due to the different chloride concentrations. Thus, the shift of the reversal potential probably causes the shift of the Po/V plot when comparing Tip-Dip data to cellular data for R29A CLIC1.

The mechanism by which the R29A mutation alters the CLIC1 open probability is unclear. In voltage gated ion channels, charged amino acids are an important component of the voltage sensing domain. At the simplest level, this can be represented by a positively charged lever that moves in response to changes in the membrane voltage, that in turn transmits force to the pore domain [38]. The substitution of the positively charged Arg29 in the CLIC1 sequence by a neutral residue (alanine) may reduce the sensitivity of CLIC1 to transmembrane voltage changes, causing the reduced open probability at voltages close to the reversal potential. However, our results from the whole cell experiments are inconsistent with this explanation because the slope of the Boltzman curve increases in R29A (Figure 7D). If Arg29 was a voltage-sensitive residue, we would expect to have a reduction in the slope (or effective charge) of the Boltzman curve, similar to that previously shown for other ion channels such as KV, NaV and CaV channels [38]. Thus, how Arg29 acts as a voltage sensor in CLIC1 is still obscure. We note that the structure of the soluble form of CLIC1 is not altered by mutating Arg29 to methionine [39], thus, we do not expect the R29A mutation to result in a structural change.

Our data demonstrate that neither Arg29 nor Lys37 affect the ion channel selectivity as the reversal potentials of R29A and K37A CLIC1 are indistinguishable from WT. Whilst we did notice small differences in the reversal potentials in the single channel currents (Figure 5) and the whole cell currents (Figure 7) of transfected HEK cells, these small differences are likely a consequence of the large variability in the intracellular chloride concentration in HEK cells. HEK cells are actively dividing and it has been demonstrated that replicating cells have different internal chloride concentration (4-20 mM) and resting membrane potentials (-30/-50 mV), corresponding to various cell shapes and various stages of the cell cycle [35].

Our results show that two separate point mutations of the only charged residues in the PTM of CLIC1 have a substantial impact on CLIC1 ion channel properties, when examined as both purified recombinant protein in Tip-Dip artificial lipid bilayers and in transfected cells. This provides strong evidence to support the view that CLIC1, at least under some circumstances, can act as an ion channel in its own right. The fact that the same electrophysiological changes occur in transfected cells and a highly purified cell-free system such as Tip-Dip argues that CLIC1 itself forms the central component of the ion channel and is not an ancillary component of an ion channel complex.
